# Recommendations for culturally safe clinical kidney care for First Nations Australians: a guideline summary

**DOI:** 10.5694/mja2.52114

**Published:** 2023-10-15

**Authors:** David J Tunnicliffe, Samantha Bateman, Melissa Arnold‐Chamney, Karen M Dwyer, Martin Howell, Azaria Gebadi, Shilpa Jesudason, Janet Kelly, Kelly Lambert, Sandawan William Majoni, Dora Oliva, Kelli J Owen, Odette Pearson, Elizabeth Rix, Ieyesha Roberts, Ro‐Anne Stirling‐Kelly, Kimberly Taylor, Gary A Wittert, Katherine Widders, Adela Yip, Jonathan Craig, Richard K Phoon

**Affiliations:** ^1^ University of Sydney Sydney NSW; ^2^ Centre for Kidney Research Children's Hospital at Westmead Sydney NSW; ^3^ University of Adelaide Adelaide SA; ^4^ Central and Northern Adelaide Renal and Transplantation Services, Central Adelaide Local Health Network Adelaide SA; ^5^ Deakin University Geelong VIC; ^6^ Kidney Health Australia Adelaide SA; ^7^ University of Wollongong Wollongong NSW; ^8^ Illawarra Health and Medical Research Institute University of Wollongong Wollongong NSW; ^9^ Royal Darwin Hospital Darwin NT; ^10^ Flinders University Adelaide SA; ^11^ Drug and Alcohol Services, South Australia Health Adelaide SA; ^12^ Central and Northern Adelaide Renal and Transplantation Royal Adelaide Hospital Adelaide SA; ^13^ Wardliparingga Aboriginal Health Equity, South Australian Health and Medical Research Institute Adelaide SA; ^14^ Cancer Research Institute University of South Australia Adelaide SA; ^15^ Southern Cross University Lismore NSW; ^16^ NSW Health Mid‐North Coast Local Health District Sydney NSW; ^17^ Aboriginal Communities and Families Health Research Alliance, South Australian Health and Medical Research Institute Adelaide SA; ^18^ Royal Adelaide Hospital Adelaide SA; ^19^ Westmead Hospital Sydney NSW

**Keywords:** Guidelines as topic, Kidney diseases, Renal dialysis, Health services, Social determinants of health

## Abstract

**Introduction:**

First Nations Australians display remarkable strength and resilience despite the intergenerational impacts of ongoing colonisation. The continuing disadvantage is evident in the higher incidence, prevalence, morbidity and mortality of chronic kidney disease (CKD) among First Nations Australians. Nationwide community consultation (Kidney Health Australia, Yarning Kidneys, and Lowitja Institute, Catching Some Air) identified priority issues for guideline development. These guidelines uniquely prioritised the knowledge of the community, alongside relevant evidence using an adapted GRADE Evidence to Decision framework to develop specific recommendations for the management of CKD among First Nations Australians.

**Main recommendations:**

These guidelines explicitly state that health systems have to measure, monitor and evaluate institutional racism and link it to cultural safety training, as well as increase community and family involvement in clinical care and equitable transport and accommodation. The guidelines recommend earlier CKD screening criteria (age ≥ 18 years) and referral to specialists services with earlier criteria of kidney function (eg, estimated glomerular filtration rate [eGFR], ≤ 45 mL/min/1.73 m^2^, and a sustained decrease in eGFR, > 10 mL/min/1.73 m^2^ per year) compared with the general population.

**Changes in management as result of the guidelines:**

Our recommendations prioritise health care service delivery changes to address institutional racism and ensure meaningful cultural safety training. Earlier detection of CKD and referral to nephrologists for First Nations Australians has been recommended to ensure timely implementation to preserve kidney function given the excess burden of disease. Finally, the importance of community with the recognition of involvement in all aspects and stages of treatment together with increased access to care on Country, particularly in rural and remote locations, including dialysis services.

First Nations peoples have inhabited Australia for at least 65 000 years.^1^ First Nations Australians have displayed strength and resilience in the face of the ongoing colonisation of Australia, fragmented communities, and loss of culture, language and connection to Country. The resultant social injustices continue to affect the determinants of health, leading to subsequent chronic disease.^2^, ^3^ Compared with non‐Indigenous Australians, First Nations Australians currently experience lower incomes, lower education attainment, lower rates of home ownership, and higher rates of unemployment and imprisonment.^4^

The incidence, prevalence and burden of chronic kidney disease (CKD) in First Nations Australians is one of the highest in the world, which is reflective of the social gradient of disadvantage.^5^, ^6^, ^7^, ^8^ First Nations Australians experience two times higher CKD prevalence^9^ and eight to nine times higher rates of kidney replacement therapy than the non‐Indigenous Australian population.^10^ These disparities demonstrate the impacts of social disadvantage without evidence of genetic predisposition. Addressing the social determinants of health to achieve equity has been emphasised by the World Health Organization^3^ and underpins chronic disease prevention. It has been recognised that Closing the Gap, which aimed to improve First Nations Australians’ life expectancy, has failed to address the determinants of health that affect individuals’ health and wellbeing.^11^, ^12^ Consequently, the 2020 Closing the Gap report recommended a true partnership with First Nations Australian communities be formed when codesigning policies and programs to ensure that community needs are met.^13^ These clinical practice guidelines are the first to be developed in partnership with First Nations Australians to improve kidney health and wellbeing. The full version of the guidelines is available at www.cariguidelines.org/home/management‐of‐chronic‐kidney‐disease‐among‐first‐nations.

## Guideline scope

These guidelines aim to address the problems faced by First Nations Australians living with CKD identified through community consultations^14^, ^15^, ^16^, ^17^ and the need for recommendations identified by clinical experts.^18^ Recommendations and ungraded statements are directed towards improving clinicians’ understanding of the clinical care desired by First Nations Australians and aim to improve the equity of the management of CKD.

## Methods

These guidelines adhered to international best practice for guideline development,^19^, ^20^ including using the Grading of Recommendations, Assessment, Development and Evaluation (GRADE) approach. The Appraisal of Guidelines for Research and Evaluation (AGREE) II checklist^21^ assessment is available in the Supporting Information. The guidelines development methods are available online in Appendix A of the full guidelines.

### Community consultation

Every which way you look at renal disease in Aboriginal people, the only solutions that will work in the long term are those that are Aboriginal‐led, culturally responsive, located in Aboriginal organisations and evaluated through an Aboriginal lens. (Pat Turner CEO, National Aboriginal Community Controlled Health Organizations, National Indigenous Dialysis and Transplantation Conference, Alice Springs, Northern Territory, 2019)^22^

The broad and purposive community feedback and priority setting for these guidelines occurred during the Kidney Health Australia Yarning Kidneys,^14^ which included consultations led by Aboriginal Kidney Care Together – Improving Outcomes Now (AKction)^16^, ^17^ and Catching Some Air.^15^ A partnership approach was adopted, with a panel of clinicians involved in the care of First Nations Australians with CKD; targeted site engagement with First Nations Australian communities and services across urban, regional and remote communities; and additional consultation and feedback from national peak First Nations organisations.^23^ The 14 community consultations identified the priorities of First Nations Australians with lived experience of CKD, which informed the scope, and provided evidence to supplement studies for the development of guideline recommendations.^14^, ^15^, ^16^, ^17^ Planned ongoing engagement and consultation with targeted First Nations Australian communities throughout the guideline development were interrupted by the coronavirus disease 2019 (COVID‐19) pandemic. To overcome these challenges, group yarning sessions occurred during 2022 with community groups or, when appropriate, with local champions. The draft guideline recommendations were reviewed by 11 communities involved in the previous consultations and were consulted along with one additional consultation with another community (Box 1). The Guidelines Working Group also included First Nations peoples with lived experience of kidney disease, who provided important insights for the development of the guidelines and also informed the development of the *Flow and thrive* artwork (Box 2). All feedback was considered and the guidelines were updated accordingly (Box 3).

Box 1 **Location and details of targeted community consultations and feedback that occurred during the guidelines development**

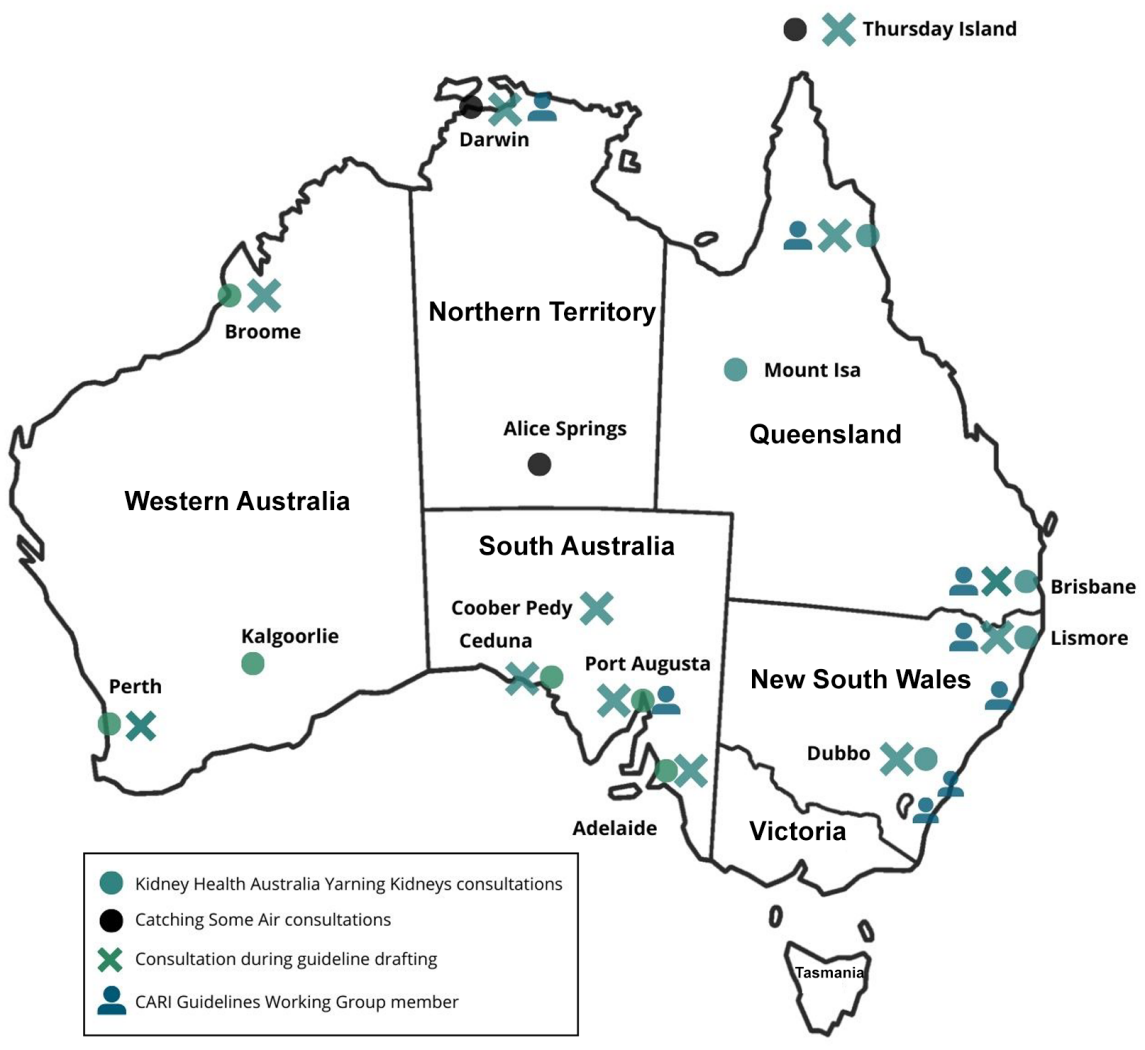

CARI = Caring for Australians and New Zealanders with Kidney Impairment.

Box 2 Flow and thrive represents the landscape of living with a kidney condition*

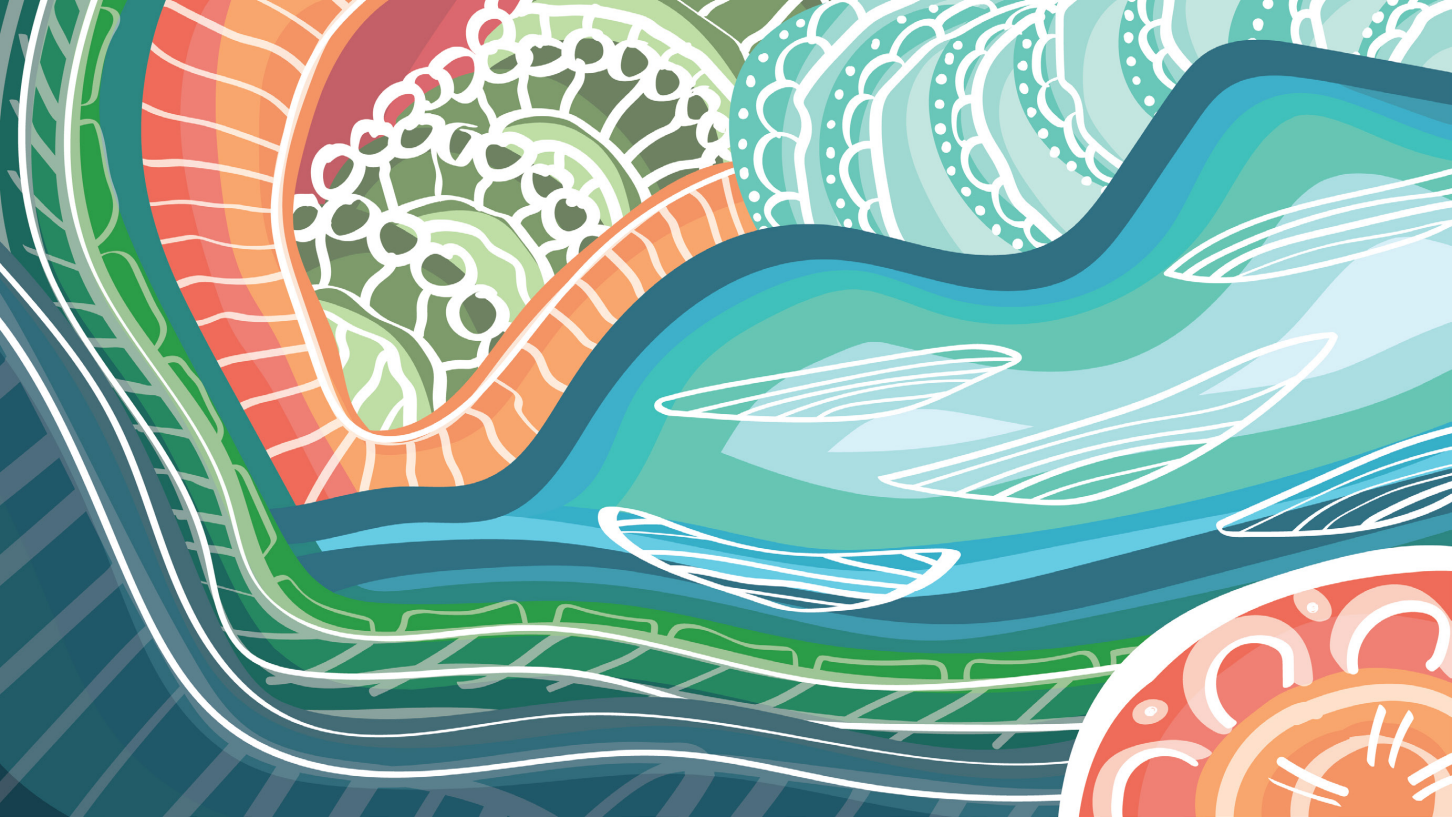

* The design represents the different stages of the health journey. At times the waters are murky and turbulent, which is represented by the tsunami on the top right of the design and the mangroves to the top left. The mangroves represent the challenges of patients moving through their varying journeys, which traverse through the rivers and into calmer seas. From the right bottom hand corner of the design, a sunrise emerges, symbolising the strength and resilience of those living with kidney conditions. This design reinforces the idea that those on this journey are never alone. The boats within the river represent the family, friends, community, and support networks that surround the patients on their wellness journey. To the far left of the design is a series of lines, which represent the various medical practitioners and clinical support personnel who guide and support the patient with their ongoing expertise and care. Image reproduced from Caring for Australians and New Zealanders with Kidney Impairment (CARI) Guidelines.^22^

Box 3 **Timeline of guidelines development**

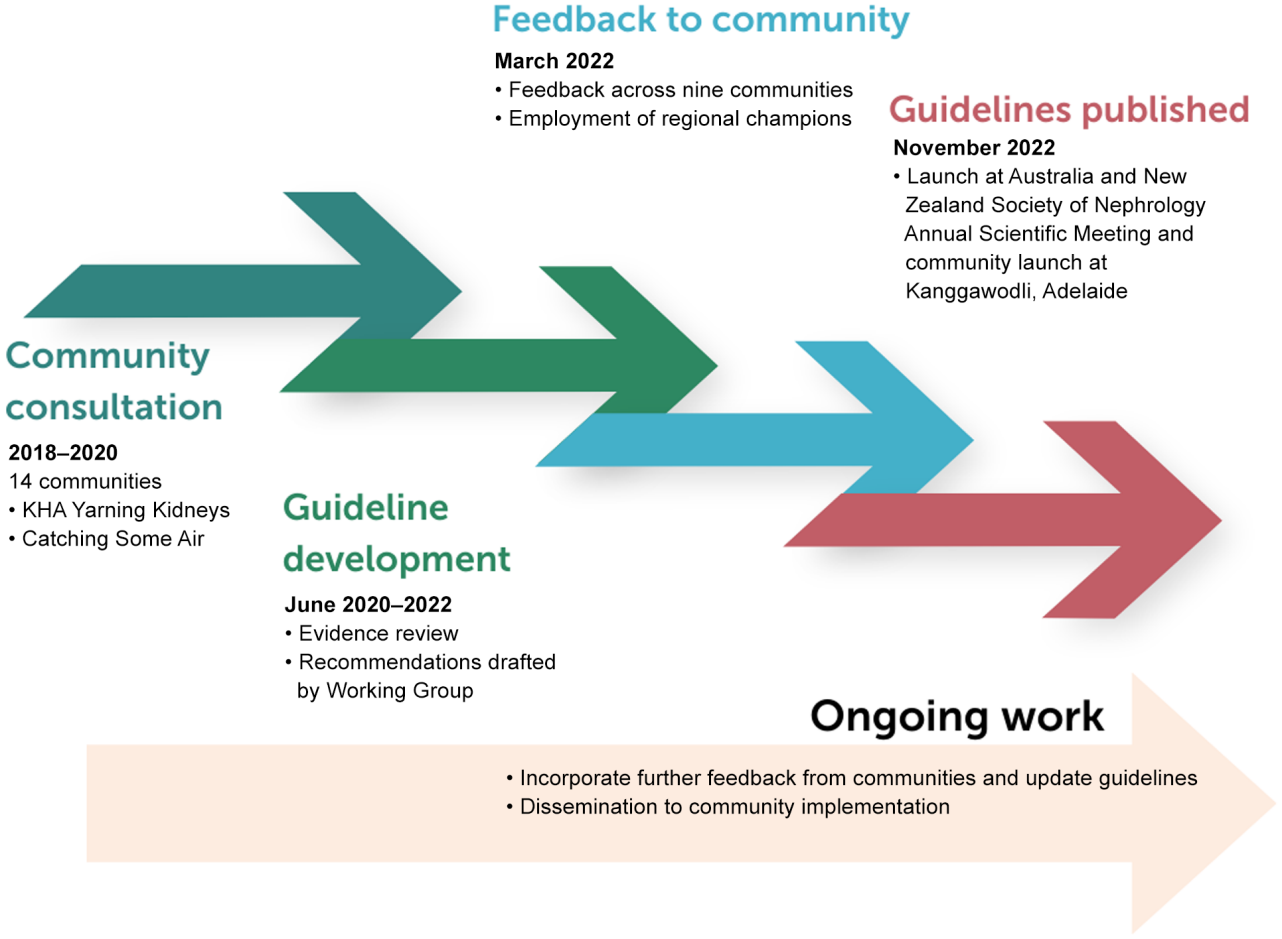

KHA = Kidney Health Australia.

### Evidence synthesis

Traditionally, clinical practice guidelines are developed with a Western biomedical view of health and have not incorporated First Nations Australian perspectives on health or health research methods, such as “yarnings”. These guidelines have extended the traditional model by including the targeted community consultation findings in the rationale for recommendations. Priorities and topics identified by the communities during the Catching Some Air^15^ and Kidney Health Australia Yarning Kidneys^14^, ^17^ consultations were transformed into focused research questions using the population intervention/exposure comparator outcome (PICO and PECO) methodology format to enable a comprehensive literature search (Appendix A in the full guidelines). There has been limited inclusion of First Nations Australians in the research priority setting of the kidney health community. As a result, we approached research questions broadly and included all study types, except for case studies and editorials, in our systematic review.

We searched MEDLINE, Embase and the Australian Indigenous Health*InfoNet* in May 2022 (Appendix B in the full guidelines). Overall, we identified 8236 citations and included 200 reports to inform the development of these guidelines (Box 4).

Box 4 **Adapted PRISMA (Preferred Reporting Items for Systematic Reviews and Meta‐Analyses) 2020 flow diagram for new systematic reviews that included searches of databases and registers only**

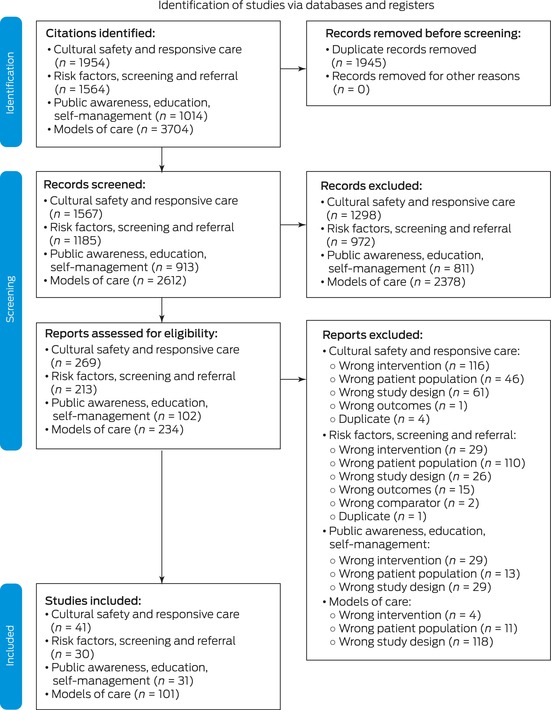

Duplicate records were identified and considered across guideline chapters. Image reproduced from Caring for Australians and New Zealanders with Kidney Impairment (CARI) Guidelines.^22^

The included studies were identified, abstracted, synthesised and appraised by two independent reviewers and approved by the Working Group. The characteristics of the included studies were summarised descriptively (Appendix C in the full guidelines), and findings across studies were combined using a mixed‐method synthesis approach.^24^ The certainty of the evidence was assessed using the GRADE approach for interventions and exposures^25^ and GRADE CERQual (Confidence in the Evidence from Reviews of Qualitative Research) for qualitative findings.^26^ The tables with the summary of findings were developed (Supporting Information and Appendix D in the full guidelines) and considered by the Guidelines Working Group, with expertise across First Nations health, clinical nephrology, nursing, lived experience of CKD, health economics, clinical research, guideline methodology and cultural safety. The guideline of the GRADE Evidence to Decision Framework was adapted to include the First Nations voice, with the “four Cs” of community voice: clinical evidence; cultural considerations; and cost, capacity, equity and other considerations (Box 5). The findings from the community consultations and feedback undertaken for these guidelines were considered under the “community voice” domain of the Evidence to Decision Framework.

Box 5 **“Four Cs” framework informing guideline recommendations**

**Table:** 

Domain	Description
Community voice	The priorities and knowledge of the First Nations Australian communities, as identified in the Catching Some Air and Kidney Health Australia Yarning Kidneys community consultations.
Clinical evidence	Balance of benefits and harms from the identified scientific literature from the formal systematic review undertaken for these guidelines, as well as findings from other important articles, reports, policies and documents identified outside of the systematic reviews scope. The certainty of the evidence was assessed using validated risk of bias tools and assessed using the GRADE approach according to study type (ie, quantitative, qualitative, or a combination of both).^25^, ^26^
Cultural considerations	Other important cultural phenomenon relevant to First Nations Australians that may have not been raised explicitly in the community consultation process.
Cost, capacity, equity and other considerations	The cost to the individual, the health systems, the health workforce (including the First Nations Australians health workforce) was considered. Implications of the recommendations on equity, including gender, remoteness and other markers of low socio‐economic status, were considered. Finally, the feasibility of the implementation of the guideline recommendations was included.

GRADE = Grading of Recommendations, Assessment, Development and Evaluation. Table reproduced from Caring for Australians and New Zealanders with Kidney Impairment (CARI) Guidelines.^22^

The rationale underpinning the grading and strength of the recommendations is provided in Box 6 and Box 7. The guidelines underwent peer review by experts in nephrology, First Nations health practitioners, people with lived experience of CKD, and peak First Nations organisations who were invited to provide feedback. Yarnings with First Nations communities involved in the initial community consultations and people with lived experience of CKD also provided feedback on the guidelines.

Box 6 Final grade for overall certainty of evidence*

**Table:** 

Overall evidence grade	Description
High	We are confident that the true effect lies close to that of the estimate of the effect.
Moderate	The true effect is likely to be close to the estimate of the effect, but there is a possibility that it is substantially different.
Low	The true effect may be substantially different from the estimate of the effect.
Very low	The estimate of effect is very uncertain and often will be far from the truth.

*Adapted from the Grading of Recommendations, Assessment, Development and Evaluation (GRADE) Working Group (www.gradeworkinggroup.org). Table adapted from Caring for Australians and New Zealanders with Kidney Impairment (CARI) Guidelines.^22^

Box 7 **Nomenclature and description for grading recommendations**

**Table:** 

Grade	Implications		
	Patients	Clinicians	Policy
Strong recommendation: “we recommend”	Most people in your situation would want the recommended course of action and only a small proportion would not.	Most patients should receive the recommended course of action.	The recommendation can be adopted as a policy in most situations.
Conditional recommendation: “we suggest”	The majority of people in your situation would want the recommended course of action, but many would not.	Different choices will be appropriate for different patients. Each patient needs help to arrive at a management decision consistent with their values and preferences.	The recommendation is likely to require debate and involvement of stakeholders before policy can be determined.

Table reproduced from Caring for Australians and New Zealanders with Kidney Impairment (CARI) Guidelines.^22^

## Main recommendations

### Cultural safety and responsive kidney health care

Targeted community consultations^14^, ^15^, ^16^, ^17^ identified that institutional racism continues to be experienced by First Nations Australians throughout the management of their CKD. These consultations highlighted that the provision of holistic care beyond the Western medical approach to health care is fundamental to the health of First Nations Australians living with CKD. First Nations Australians described the holistic care, including social and cultural aspects of care and involvement of family and other First Nations Australians, as integral to their overall wellbeing. Clinical practice guidelines on the management of CKD have rarely focused on psychosocial aspects of care^27^, ^28^ and, to our knowledge, have never addressed institutional racism. In recognition of the importance and impact of racism on the provision of care, the first recommendations focused on institutional racism and on providing clinical and culturally safe and responsive care for First Nations Australians. Despite the imperative of the issues identified by the community, although there are limited studies, those published have shown that interventions focused on addressing institutional racism improved cultural safety and resulted in improved access, increased self‐agency, and involvement in shared decision making among First Nations Australians^29^, ^30^, ^31^, ^32^, ^33^ (Supporting Information, tables A1 and A2, and Appendices C and D in the full guidelines). The inequalities of CKD largely result from the disproportionate impact of the social determinants of health among First Nations Australians,^6^, ^8^, ^34^, ^35^, ^36^ with very few associations of genetic alleles with CKD burden described in First Nations Australians.^36^ As a result, the Caring for Australians and New Zealanders with Kidney Impairment (CARI) Guidelines Working Group has recommended that “Indigenous status” is removed as a factor associated with CKD. The intention is to educate and place the focus on the impact of social disadvantage and to mitigate the bias and prejudice experienced by First Nations Australians, as described throughout the community consultations.^14^, ^15^, ^16^, ^17^

### Inequity and institutional racism in health care

We recommend that health services evaluate, monitor and act upon the institutional racism within their system by addressing all domains identified in the *Matrix for identifying, measuring and monitoring institutional racism within public hospitals and health services* ^37^ (*Strong recommendation*). Furthermore, we recommend that First Nations Reference Groups should be incorporated in kidney health services within Australia (*Strong recommendation*). We also recommend removing Indigenous status as a risk factor for CKD because the increased risk is explained by adverse social determinants of health, which leads to a greater burden and progression of CKD (*Strong recommendation*).

### Cultural safety

We recommend that health care staff receive effective and responsive cultural safety training, and that continuous quality improvement strategies are undertaken to address institutional racism, using tools such as the *Matrix for identifying, measuring and monitoring institutional racism within public hospitals and health services* (*Strong recommendation*).^37^

### Family‐ and community‐centred engagement and involvement in managing CKD

We recommend that the family and community of First Nations Australians with CKD are actively involved in all clinical appointments, according to individual preferences (*Strong recommendation*).

### Transport and accommodation services for First Nations Australians

We recommend that health services develop clear pathways for ensuring transport and accommodation needs are prioritised and made available to patients and their family members for all health care interactions (*Strong recommendation*).

### First Nations kidney health workforce

We recommend that health services aim to develop professional support for First Nations Australians with CKD according to their needs (*Strong recommendation*): First Nations nurses, allied health professionals, and doctors; Aboriginal Health Practitioners and/or Aboriginal Health Liaison Officers; patient preceptors/navigators; and interpreters.

### Screening, and referral of CKD

Early targeted screening is an important and cost‐effective strategy to prevent the occurrence and progression of CKD and reduce cardiovascular disease.^38^, ^39^ Concerted efforts to decrease late referrals (less than three months between referral and the commencement of kidney replacement therapy) among First Nations Australians have led to improvements in earlier engagement with specialists’ services.^40^ The guidelines indicate that “Indigenous status” is not an independent factor associated with CKD, the Working Group recommends earlier screening and referral criteria for First Nations Australians. This recommendation is in response to the substantially higher incidence, more rapid progression, and excess burden of disease,^9^, ^10^ and the findings from the community consultations.^14^, ^15^, ^16^, ^17^

### Identification of factors associated with CKD in First Nations Australians

The ungraded statements highlight that an individual's susceptibility to CKD (Box 8) is increased by the following factors: family history of CKD, diabetes mellitus, hypertension, obesity, established cardiovascular disease, history of acute kidney injury, and cigarette smoking. Additional considerations include history of low birthweight, history of recurrent childhood infections, remoteness, low socio‐economic status, housing insecurity and overcrowding, education levels, and other impacts of colonisation.

Box 8 **Factors associated with chronic kidney disease among First Nations Australians**

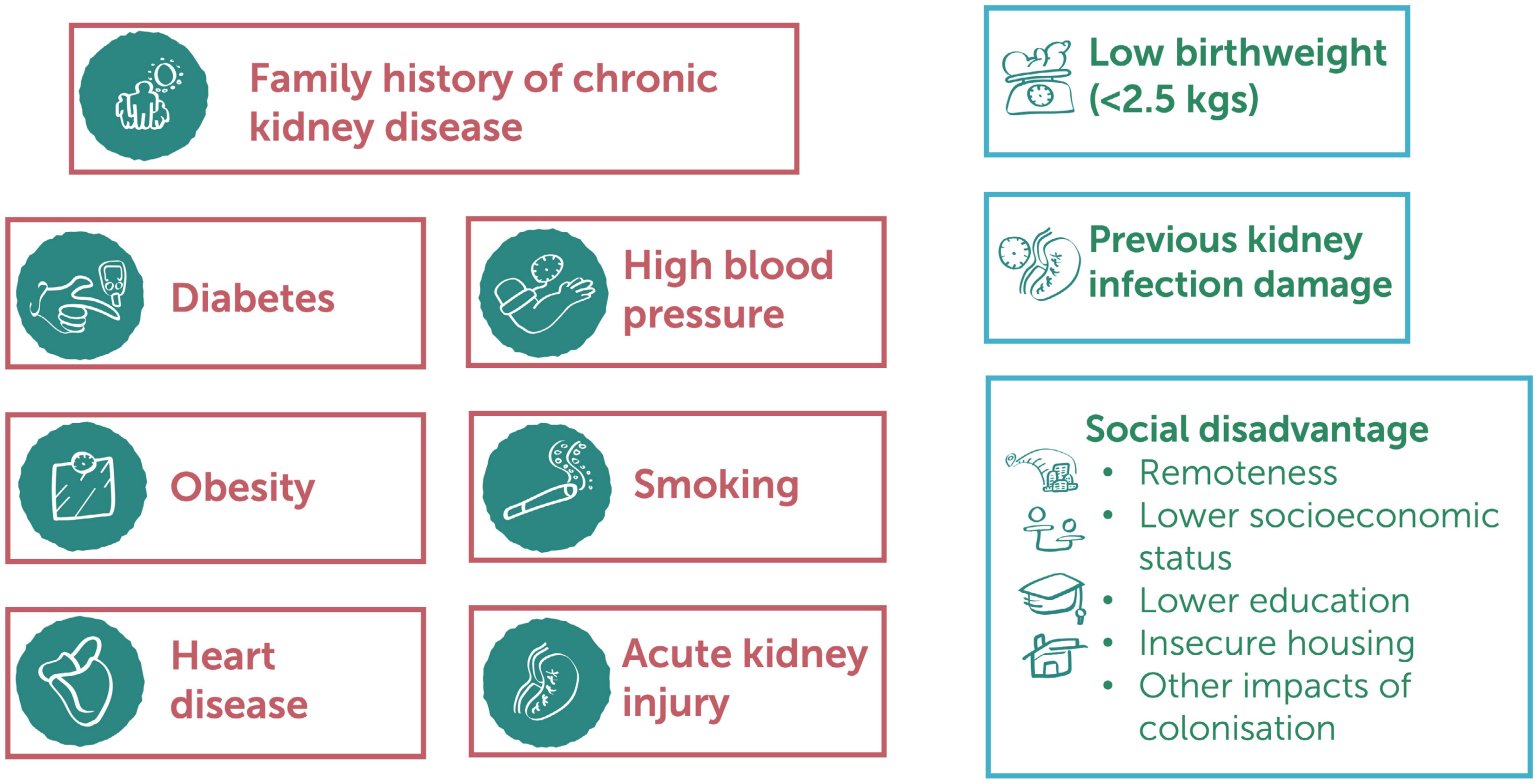

Image reproduced from Caring for Australians and New Zealanders with Kidney Impairment (CARI) Guidelines.^22^

### Screening and early detection programs for CKD among First Nations Australians

We recommend that First Nations Australians receive age‐appropriate health assessments to screen for CKD at least annually, including using the specific Medicare item number for Aboriginal and Torres Strait Islander peoples health assessment (Box 9) (*Strong recommendation*).

Box 9 **Chronic kidney disease (CKD) screening matrix for First Nations Australians**

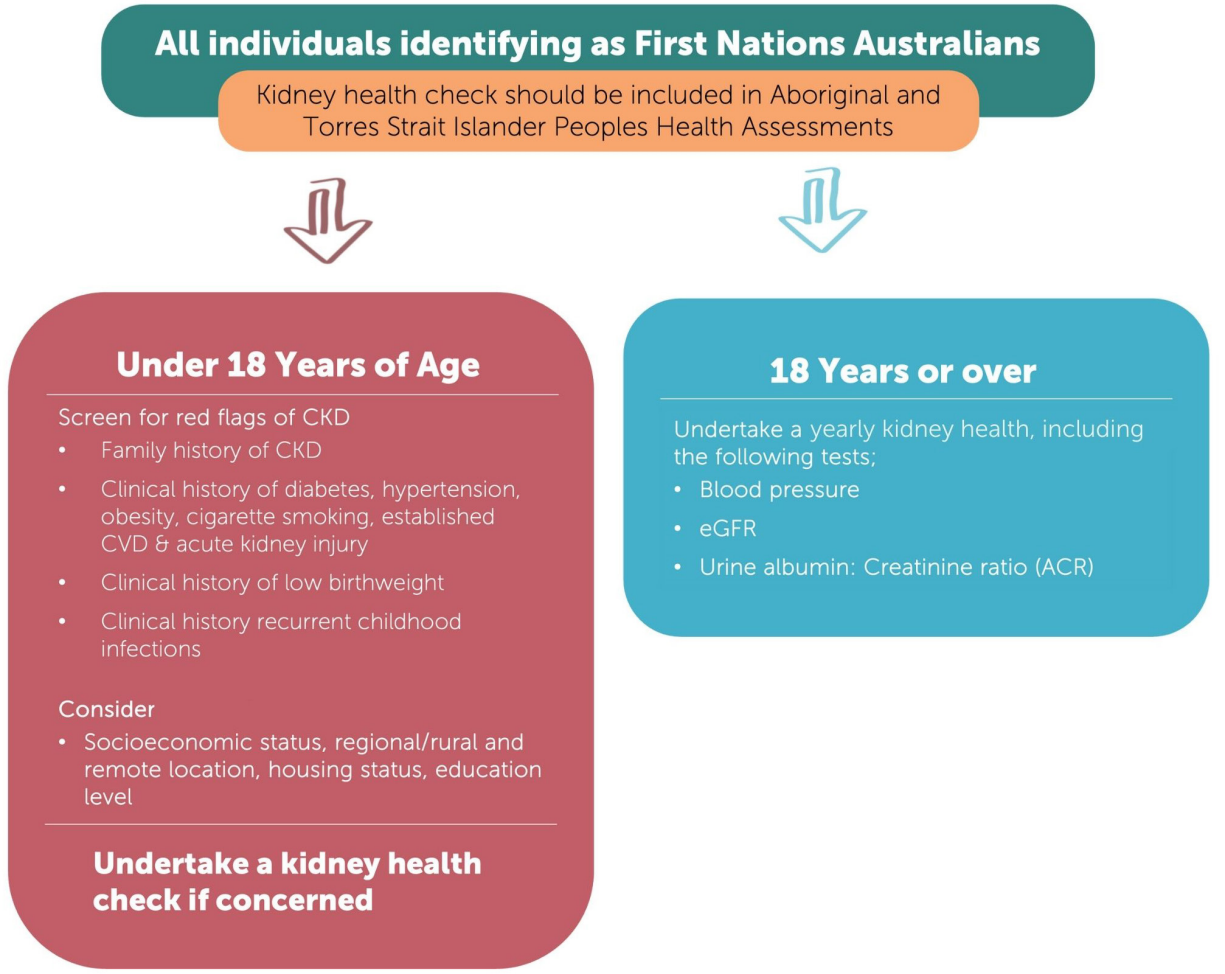

CVD = cardiovascular disease; eGFR = estimated glomerular filtration rate. Image reproduced from Caring for Australians and New Zealanders with Kidney Impairment (CARI) Guidelines.^22^

### Referral practices for First Nations Australians with CKD

We suggest that referrals of First Nations Australians with CKD to nephrologists (or specialist‐supported kidney health teams) should be done using one or more of the following criteria (Box 10) (*Conditional recommendation*):

- eGFR ≤ 45 mL/min/1.73 m^2^;
- persistent significant albuminuria > 30 mg/mmol;
- a sustained decrease in eGFR > 10 mL/min/1.73 m^2^ per year; and
- elevated blood pressure that is not within the target range despite the use of at least three antihypertensive agents.

Box 10 **Referral matrix for First Nations Australians**

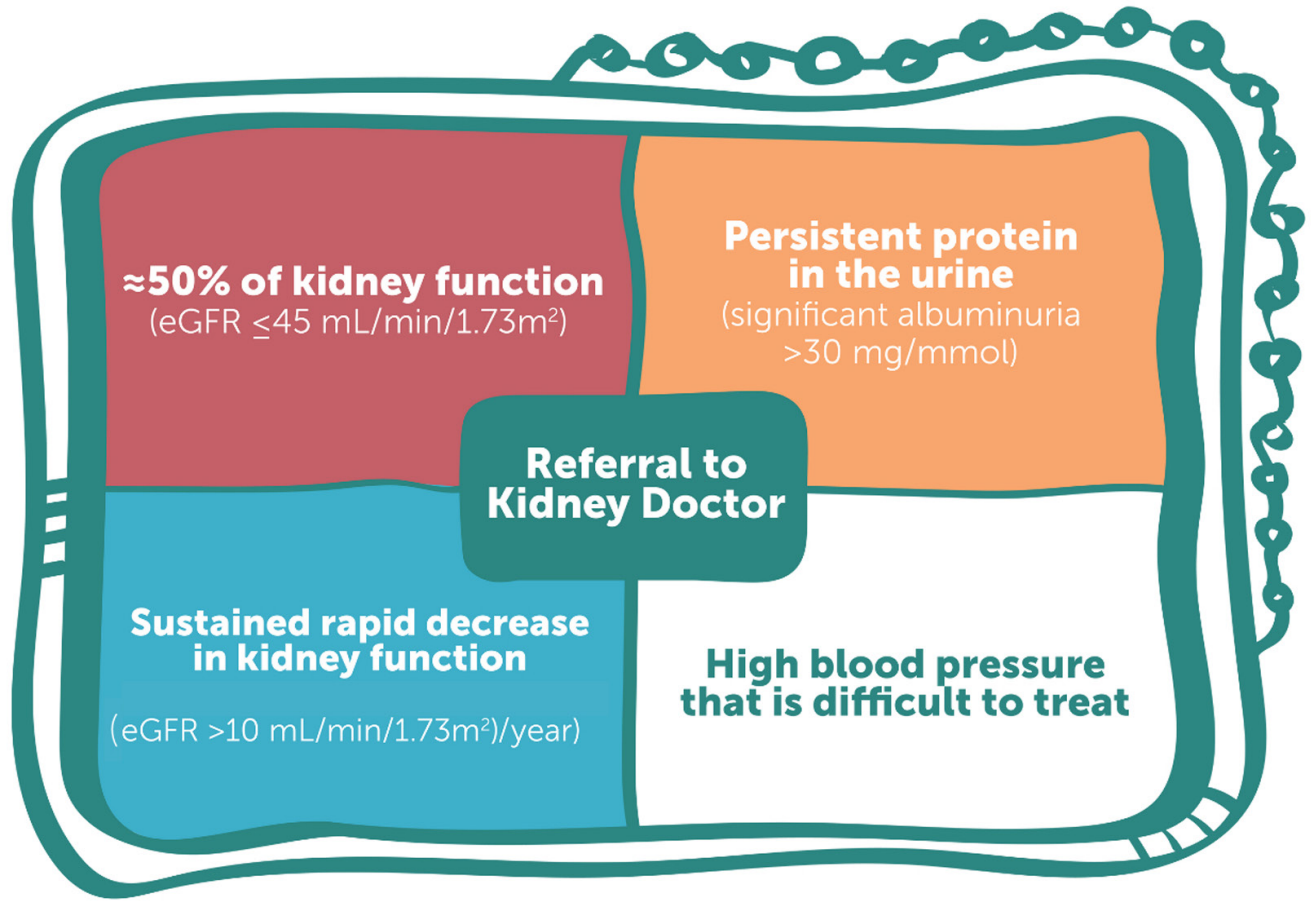

eGFR = estimated glomerular filtration rate. Image reproduced from Caring for Australians and New Zealanders with Kidney Impairment (CARI) Guidelines.^22^

### Public awareness, education and self‐management

Targeted community consultations emphasised the need for greater awareness and education to prevent and manage CKD. There was a desire for further engagement of local communities to deliver tailored community‐based awareness and education. Supporting First Nations Australians to self‐manage their disease, including incorporating local bush tucker, bush medicine, and other cultural activities was also a priority.^14^, ^15^, ^16^, ^17^ Despite the limited published scientific evidence, these guidelines provide recommendations focused on increased community ownership and incorporation of First Nations culture and activities within CKD education and self‐management initiatives. The guideline recommendations and ungraded statements for this section are summarised in Box 11.

Box 11 **Additional recommendations and ungraded statements for Caring for Australians and New Zealanders with Kidney Impairment (CARI) Guidelines management of chronic kidney disease (CKD) among First Nations Australians^22^**

**Table:** 

Guideline chapter	Recommendations and ungraded statement
**Cultural safety and responsive kidney health care**	
Inequity and institutional racism in health care	Social determinants of health associated with lower kidney function among First Nations Australians include (but are not limited to): ‣ low socio‐economic status; ‣ living in a remote area; ‣ housing insecurity; ‣ unemployment; ‣ surviving on welfare; ‣ poor food security. Institutional racism could be addressed by actively decolonising services by starting with: ‣ exercising critical reflexivity; ‣ recognising and addressing power imbalances; ‣ enabling reciprocity; ‣ transformative practice ‣ fostering two‐way communication and understanding between clinicians and patients and their families; ‣ increasing access to culturally shaped care; ‣ increasing self‐determination and empowerment of patients, families and communities forced to engage with mainstream kidney health services. A confidential, culturally appropriate complaints process should be established to enable patients, families and community members to provide feedback on their experiences regarding all kidney health services in Australia.
Cultural safety	We recommend that patients and family members are a part of the design and delivery of cultural safety training for health care staff, which includes using Indigenous ways of knowing, being and doing, for example, informal Yarning style communication (*Strong recommendation*). General: ‣ First Nations Australians with CKD and their family members should be recognised as experts in their own lives, health care needs, and priorities; ‣ First Nations Australians’ connection to culture and community should be actively supported, as it contributes to, and is protective for, their health and wellbeing; ‣ cultural, family and community commitments and priorities should be assigned equal importance to clinical care considerations for First Nations Australians; ‣ culturally safe care is determined by the recipient of care. Cultural safety training: ‣ providing culturally safe care is a lifelong journey involving ongoing critical self‐reflection for health care professionals and a supportive work environment; ‣ health services are responsible for mandating and delivering effective, targeted and sustainable cultural safety training that actively addresses unconscious bias and interpersonal and systemic racism, delivered in a non‐threatening and supportive workplace; ‣ all cultural safety training should be evaluated for effectiveness, and opportunities actively identified to improve its delivery and implementation;
Family and community centred engagement and involvement in managing CKD	Health systems should facilitate appropriate mechanisms to support family members to accompany First Nations Australians who are forced to relocate for the management of CKD. Psychological and social support should be made available for family members supporting First Nations Australians with CKD. Family and community should be included in decision making regarding dialysis and transplantation, in line with the First Nations kinship systems and patients’ preferences.
Transport and accommodation services for First Nations Australians	No additional recommendations and ungraded statements.
First Nations health workforce	First Nations health practitioners should receive formal education and training in CKD, treatment options and management. Further financial investment and strategic commitment to implement, evaluate and upscale a multidisciplinary, community‐based First Nations health workforce is required.
**Screening, and referral of CKD**	
Identification of factors associated with CKD progression in First Nations Australians	No additional recommendations or ungraded statements.
Screening and early detection programs for CKD among First Nations Australians	We suggest that CKD screening programs of First Nations Australians are community‐controlled, codesigned and use an integrated multidisciplinary approach (*Conditional recommendation*). Screening for CKD among First Nations Australians should occur at least annually. The urine albumin to creatinine ratio measurement in a first void specimen for the detection of proteinuria is preferred, but a random spot urine specimen may be adequate when conducting opportunistic testing. The diagnosis of persistently elevated urine albumin to creatinine ratio requires at least two measurements over three months.
Referral practices for First Nations Australians with CKD	No ungraded statements.
**Public awareness, education and self‐management**	
Public awareness — to enable First Nations Australians to access information about kidney disease before screening and diagnosis	We recommend that all health promotion and public awareness campaigns be codesigned with community and include First Nations Australians with lived experience of kidney disease (*Strong recommendation*). We suggest that health services co‐create health promotion and public awareness tools with First Nations Australians to highlight risk factors for CKD using Indigenous methods and a range of resources and information, including the sharing of stories in community settings (*Conditional recommendation*). No ungraded statements
Education to support engagement and treatment	We recommend that educational initiatives that facilitate engagement and self‐efficacy in management of CKD are community‐led and codesigned (*Strong recommendation*). Education resources and programs should be translated and interpreted to local languages, to actively address the language barriers. Education resources and programs should be included as part of a multifactorial approach to managing CKD in First Nations Australians. Education should encompass a range of techniques and programs, and include elements such as: ‣ yarning circles and storytelling; ‣ involvement of Elders and First Nations Australians with lived experience of kidney disease; ‣ interactive lectures and videos; ‣ cooking classes, shopping classes, and/or community gardens; ‣ art and music.
Self‐management programs and initiatives	We recommend self‐management support (initiatives and programs) should be (*Strong recommendation*): ‣ available to patients across all stages of CKD (not exclusive to patients undergoing dialysis) and initiated upon initial engagement with health care services; ‣ ongoing and collaborative, use a whole‐person approach, and acknowledge the roles and responsibilities of both patients and health care providers and the reciprocal interactions between the intra‐ and interpersonal and institutional domains; ‣ facilitate access to multidisciplinary input (including exercise physiology, psychology, dietetics, audiology, podiatry, physiotherapy, optometry, and oral health/dental services) according to the individual's needs. We recommend that adequate, sustainable and long term funding models be developed and implemented to support the codesign, development, implementation and continuation of self‐management and behaviour change programs (*Strong recommendation*). Culturally appropriate self‐management support is necessary for an individual's social, cultural and emotional wellbeing. Self‐management support and strategies should: ‣ be culturally safe, personalised, contextual and holistic (social, physical, psychological and spiritual); ‣ focus on social and emotional wellbeing, including connection to Country, community and family; ‣ ensure that time and effort is taken to understand the individual, their social context, to develop a trusting clinician–patient relationship — this relationship building should employ active listening strategies and use a non‐stigmatising approach; ‣ ensure that individual independence and autonomy is maintained; ‣ include the establishing of peer story‐sharing programs and resources to support self‐management and behaviour change across all stages of CKD. Self‐management programs need to be consistent, particularly regarding the information and advice about diet and nutrition for CKD (see CARI commentary on the KDOQI nutrition guidelines^41^), and occur within a framework of First Nations food knowledge and customs. The broader elements influencing eating behaviours and food security, such as availability, accessibility, affordability, and facilities for storage and preparation, should also be acknowledged and addressed.
**Models of care**	
Models of care: CKD (pre‐dialysis)	We suggest maintaining clinical and medical information systems (technology, programming, documentation) according to appropriate data sovereignty principles, ongoing quality improvement tools and program evaluation to support program delivery, audit and evaluation (*Conditional recommendation*). No ungraded statements.
Models of care: CKD (kidney failure)	We suggest that First Nations Australians have access to dedicated multidisciplinary teams for the management of kidney replacement therapy, including, where available, First Nations doctors and/or nurses, Aboriginal Liaison Officers, Aboriginal Health Practitioners and/or First Nations peer navigators (*Conditional recommendation*). There is insufficient evidence to recommend a standard approach for vascular access choice or management. A patient‐centred approach based on local practices should be adopted and undertaken.
Models of care: transplantation	Kidney transplantation should be considered and discussed as a treatment option for all First Nations Australians with CKD requiring kidney replacement therapy. There are ongoing studies regarding the most appropriate and effective models of care for transplantation that will inform the future development of guideline recommendations.

CARI = Caring for Australians and New Zealanders with Kidney Impairment; KDOQI = Kidney Disease Outcomes Quality Initiative.

### Models of care

A higher proportion of First Nations Australians live in rural and remote locations compared with non‐Indigenous Australians.^42^ Therefore, the management of CKD in First Nations Australians often requires extensive travel and potential relocation for extended periods, particularly for kidney replacement therapy.^43^ Relocation for treatment significantly affects people with CKD and their families,^44^, ^45^ including high out‐of‐pocket expenses and dislocation from family, community, culture and Country.^14^, ^15^, ^16^, ^17^ These guidelines provide recommendations on the structure and components of how care should be delivered for First Nations Australians with CKD, with a focus on keeping people on Country where possible. Recommendations for the models of care for transplantation will be updated with the completion of studies such as the National Indigenous Kidney Transplant Taskforce^46^, ^47^, ^48^ and Return to Country.^49^

### Models of care in CKD (pre‐dialysis)

We recommend the development of dedicated programs specific for First Nations Australians to identify people at risk and diagnose and manage CKD (*Strong recommendation*).

We recommend that these programs (*Strong recommendation*):

- are codesigned and governed by First Nations communities and embedded into existing chronic disease programs;
- are adapted to ensure they are culturally safe, tailored to the community and flexible to the changing needs of the community; and
- explicitly identify and address barriers to care, including (but not limited to) institutional racism, cultural barriers, social and/or geographical barriers, transportation and/or other costs of health care to patients and families.

We suggest that programs of care (*Conditional recommendation*):

- be conducted within community‐controlled health services;
- use a multidisciplinary approach, with program‐specific health care workers dedicated to facilitating these programs;
- promote a culturally safe workplace culture with built‐in support for the multidisciplinary team, including (but not limited to) the provision of continuing education programs, case management workshops, clinic guidelines/protocols, and online educational materials;
- incorporate patient education and training within the program to increase patient and community empowerment; and
- use an integrated care approach when specialised nephrology services are required as part of a patient's management, including the use of telehealth services where appropriate.

### Models of care in kidney failure

We recommend that health services work in partnership with Aboriginal community‐controlled health organisations to establish community‐controlled models of care that address local needs and conditions. This will allow First Nations Australians to have nurse‐supported and/or Aboriginal Health Practitioner‐supported dialysis on Country, particularly in remote areas (*Strong recommendation*). Furthermore, we recommend that kidney health services incorporate dialysis services (eg, mobile dialysis) to facilitate patients to have dialysis in locations where it is not otherwise available. This service should include adequate transport, accommodation, and workforce support to ensure equity (*Strong recommendation*).

We recommend the availability of First Nations Australians‐specific and codesigned programs to address the social and emotional wellbeing of people undergoing kidney replacement therapy (*Strong recommendation*). We suggest supported home dialysis models, such as community‐based home dialysis, to allow First Nations Australians to access the benefits of home dialysis (*Conditional recommendation*).

We recommend that kidney health services use telehealth/video link models to augment face‐to‐face care for those living and receiving dialysis in regional and remote areas (*Strong recommendation*). Box 11 lists other guideline recommendations not detailed in the guideline summary.

### Suggestions for future research

Future research should be led by and in partnership with First Nations Australian communities and to the standards set out in the Aboriginal and Torres Strait Islander Quality Appraisal Tool^50^ and reported to the standards of the Consolidated Criteria for Strengthening the Reporting of Health Research Involving Indigenous Peoples (CONSIDER) Statement.^51^ Undertaking true collaborative approaches will ensure that research is directed on the communities’ priorities and conducted correctly. For example, future research priorities from the CARI Guidelines Working Group include evaluating patient preceptor programs in CKD care for First Nations Australians and further evaluating nurse‐supported dialysis models of care within communities.

## Conclusions

These clinical practice guidelines represent a major first step in addressing the evident disparity in CKD among First Nations Australians compared with non‐Indigenous people in Australia^9^, ^10^ and improve cultural safety in health systems. Importantly, the guidelines have been developed in partnership with First Nations Australians, prioritising the Community Voice, and provide opportunity for the crucial next step of implementation.

We have done talking, it is time to implement, to change, let's do it now. (Kelli Owen, CARI First Nations Australians CKD Guidelines Launch, 16 October 2022, Sydney, New South Wales).^52^

Ensuring that the guidelines are drivers of equity in the health system will require ongoing partnership with First Nations communities. The evaluation of these guidelines needs to be led by First Nations Australians as well as providing transparent accountability in their implementation across nephrology units in Australia.

## Open access

Open access publishing facilitated by The University of Sydney, as part of the Wiley – The University of Sydney agreement via the Council of Australian University Librarians.

## Competing interests

Karen Dwyer is the current Clinical Director of Kidney Health Australia and Chair of the Clinical Advisory Board and has received consultancy fees, honoraria and research funding from GSK, Servier, Bayer and Novartis; and research funding from Genzyme, A2 Milk Company, ROTRF, CellCept Australia, and Amgen; and is on the clinical advisory board of GMHBA. Gary Wittert has received funding from the Australian Health Practitioner Regulation Agency for medico‐legal reports; honoraria from Elsevier for journal editor and international advisory board roles; honoraria from Bayer for an advisory board role; and research funding from Bayer and Lilly. Richard Phoon has received consultancy fees and honoraria from AstraZeneca, Novo Nordisk and Sanofi–Genzyme, and travel support from Novartis. All these potential conflicts have little to no relevance to these guidelines.

## Provenance

Not commissioned; externally peer reviewed.

## Supporting information


Supporting Information
