# Supplementary material for: Recommendations for culturally safe clinical kidney care for First Nations Australians: a guideline summary
Source: Med J Aust. 2023 Oct 15;219(8):374–85. doi: 10.5694/mja2.52114 (PMC10952490; doi:10.5694/mja2.52114)
Supplement: Supplementary file 1 — Supporting Information [file MJA2-219-374-s001.pdf]

The Medical Journal of Australia

Supporting Information

Supplementary material

**This appendix was part of the submitted manuscript and has been peer reviewed.
It is posted as supplied by the authors.**

Appendix to: Tunnicliffe DJ, Bateman S, Arnold-Chamney M, et al. Recommendations for culturally safe and clinical kidney care for First Nations Australians: a guideline summary. *Med J Aust* 2023; doi: 10.5694/mja2.52114.

APPENDIX

CULTURALLY SAFE CLINICAL KIDNEY CARE GUIDELINES - Compliance with AGREE II reporting checklist

(https://www.agreetrust.org/resource-centre/agree-reporting-checklist/)

Domain 1: Scope and Purpose

Objectives:

- To ensure clinical practice aligns with the best available evidence;
- To improve the identification, management of CKD among First Nations Australians with CKD
- To provide recommendations on improved culturally safe and responsive care for First Nations Australians with CKD

Questions: Research questions to determine scope of the guideline were developed by the guideline writing group comprised of nephrologists, endocrinologist, renal nurses, health economists, guideline methodologists, people with lived experience with kidney disease and informed by a nationwide community consultation. Further details of the research questions (PICOM/PECOM format) address in these guidelines are detailed in Table A1 available in Appendix A (https://www.cariguidelines.org/first-nations-australians-guidelines-appendix-a/) of the full guidelines.

Domain 2: Stakeholder Involvement

Group membership: The guideline writing group consisted of five First Nations Australians researchers in Indigenous Health, four First Nations Australians with lived experience of kidney disease, six nephrologists, one endocrinologist, three renal nurses and researchers, one renal dietitian and researcher, one health economists, three guideline methodologists CARI Guidelines office.

- *Convenor*: Richard Phoon, Nephrologist and Clinical Researcher. Westmead Hospital, The University of Sydney, Sydney, NSW, Australia
- *First Nations Australians Researchers*: Dora Oliva, South Australian Health, SA; Kelli J Owen, Royal Adelaide Hospital, University of Adelaide, Adelaide, SA; Odette Pearson and Kimberly Taylor, South Australian Health and Medical Research Institute, SA, Australia; Ro-Anne Stirling Kelly; The Children's Hospital at Westmead, Sydney, NSW, The University of Sydney, Sydney, NSW, Mid-North Coast Local Health District, NSW Health, NSW.
- *First Nations Australians with lived experience of kidney disease*: Azaria Gebadi, Ieyesha Roberts, and Katherine Widders, The Children's Hospital at Westmead, Sydney, NSW, The University of Sydney, Sydney, NSW; Kelli J Owen, Royal Adelaide Hospital, University of Adelaide, Adelaide, SA.
- *Nephrologists and Clinical Researcher*: Samantha Bateman, Royal Adelaide Hospital, University of Adelaide, Adelaide, SA; Karen Dwyer, Deakin University, Kidney Health Australia, Melbourne, VIC; Shilpanjali Jesudason, Royal Adelaide Hospital, University of Adelaide, Adelaide, SA; Sandawan William Majoni, Royal Darwin Hospital, Flinders University, Menzies School of Health Research, Darwin, NT; Jonathan C Craig, Flinders University, SA.
- *Endocrinologist and Clinical Researcher:* Gary A Witter, University of Adelaide, SA.

- *Renal Nurses and Clinical Researchers:* Melissa Arnold-Ujvari, Janet Kelly, The University of Adelaide, SA, Liz Rix, Southern Cross University, QLD, The University of Adelaide, SA
- *Renal dietitian and Researcher:* Kelly Lambert, University of Wollongong, NSW, Illawarra Health and Medical Research Institute, NSW
- *Health Economists:* Martin Howell, The Children's Hospital at Westmead, The University of Sydney, Sydney, NSW
- *Guideline methodologists:* Martin Howell, The Children's Hospital at Westmead, The University of Sydney, Sydney, NSW; David J Tunnicliffe, The Children's Hospital at Westmead, The University of Sydney, Sydney, NSW; Adela Yip The Children's Hospital at Westmead, The University of Sydney, Sydney, NSW, Search strategies and evidence synthesis for all guideline subtopics.

Target population preferences and views: The Kidney Health Australia (KHA) Yarning Kidneys(1-3) and Catching Some Air(4) informed scope of the guidelines and the development of guideline recommendations. The literature searches included qualitative studies addressing patient and caregiver perspectives. The Work Group also included First Nations Australians with lived experience of kidney disease. CARI Guidelines also feedback to communities involved in the initial community consultation process to ensure the patient preferences and views were considered and addressed in the guidelines.

Target users: The guidelines may be used by healthcare workers caring for First Nations Australians with chronic kidney disease. The guidelines are also aimed at health systems and health policy to inform standards of care and clinical decisions related to holistic care for First Nations Australians with kidney disease. Health professionals, and Indigenous Health Experts were involved in the peer review of the guidelines, and invited to provide comment and feedback in the draft through professional societies, and peak organisations including the Australian and New Zealand Society of Nephrology, the Renal Society of Australia, Transplant Society of Australia and New Zealand, National Aboriginal Community Controlled Health Organisation, Purple House, Royal Australian College of General Practitioners, Royal College of Physicians, The Congress of Aboriginal and Torres Strait Islander Nurses and Midwives, and National Association of Aboriginal and Torres Strait Islander Health Workers and Practitioners. The comments were discussed by the writing group and used to update and improve the evidence for the recommendations.

Domain 3: Rigour of development

Search methods: Search methods for each subtopic, including a full search strategy are reported and available in Appendix B (https://www.cariguidelines.org/first-nations-australians-guidelines-appendix-b/).

Evidence selection criteria: All study designs, comparisons and outcomes for studies were included and detailed in the Table A1 available in Appendix A (https://www.cariguidelines.org/first-nations-australians-guidelines-appendix-a/) of the full guidelines.

Strengths and Limitations of the evidence: Critical appraisal of the evidence is reported in the evidence tables (Appendix C; https://www.cariguidelines.org/first-nations-australians-guidelines-appendix-c/). A Summary of the Findings Tables for each topic is reported in Appendix D (https://www.cariguidelines.org/first-nations-australians-guidelines-appendix-d/)

for the full guidelines. The adapted evidence to decision framework (Four C's) includes discussion of benefits and harms from the clinical evidence as well as the limitations of the data using the GRADE Assessment.

Formulations of recommendations: Recommendations were formulated based on an adapted GRADE Evidence to Decision Framework to include the community voice and other cultural Safety implications. The full guidelines detail each aspects of the Evidence to Decision Framework in detail for all guideline recommendations.

Consideration of benefits and harms: Clinical evidence is a domain of the Four C's Evidence to Decision Framework.

Link between recommendations and evidence: The evidence for each subtopic was systematically screened, extracted and summarised with appraisal, and recommendations used the GRADE Assessment to qualify the certainty of the evidence in relation to the recommendation. Evidence tables are provided for each guideline subtopic.

External Review: Six peer reviewers with expertise in the subtopic/field were identified by the Work Group (including nephrologists, Indigenous Health) from hospitals and Universities around Australia. Reviewers were invited to complete a review form, and/or provide comment directly on the draft guideline manuscript. Final drafts were posted for public review for one month. Comments, suggestions and feedback were integrated into the final versions of the manuscript.

Updating procedure: This guideline will be reviewed and updated to account for new evidence relating to the topic that may:

- Warrant inclusion of additional recommendations;
- Alter the strength of a recommendation, i.e. upgrade a suggestion to a recommendation or conversely downgrade a recommendation to a suggestion.
- Warrant removal or change to a recommendation or suggestion;

There is no set timeline for updating, but the Working Group is committed to providing recommendations on transplantation upon the completion of the National Indigenous Transplant Taskforce studies and Return to Country Study.

Domain 4: Clarity of Presentation

Graded recommendations and suggestions are provided in a box at the beginning of each guideline subtopic, with reference to the population or subgroup where relevant. Where there is uncertainty in the evidence, this is reflected in the grade of the evidence available with the full guideline and may be supported by ungraded statements.

Domain 5: Applicability

Facilitators and barriers to application: These guidelines are based on the best available evidence, with consideration from the authors regarding potential barriers to application such as cost and time, and facilitators such as current practice. Further relevant details may be discussed within guideline subtopics.

Implementation advice/tools: Each guideline subtopic contains suggestions for implementation, and future research.

Resource implications: Each guideline recommendation includes Cost, capacity, equity and other considerations as a domain for the Four C's. Cost-effectiveness studies were included in the search and other implications for costs are discussed to inform guideline recommendation development.

Monitoring auditing criteria: Where relevant, the guidelines recommend audit criteria or contain suggestions for audit are discussed in the Cost, capacity, equity and other consideration domain. An overall statement about implementation and audit is provided in the guidelines

Domain 6: Editorial independence

The development of the CARI guideline for Recommendations for culturally safe kidney care for First Nations Australians was funded by Kidney Health Australia, who received a grant from the Federal Government. The funding bodies did not influence the content or scope of the guideline.

Competing interests: The authors have no competing interests to declare as identified by the CARI Guidelines conflict of interest form completed by all authors and detailed in the guideline summary and the full guidelines. The conflict of interest form adheres to the NHMRC Guidelines to Guidelines standards. A conflict of interest statement for all authors is provided in the guidelines and the guideline summary.

Summary of finding tables

Please see appendix of the full guidelines for details on the rating certainty of the evidence for each summary of finding available at the CARI Guidelines website (https://www.cariguidelines.org/first-nations-australians-guidelines-appendix-d)

Cultural safety and responsive kidney health care

Table A1. Summary of findings – Interventions for addressing inequity and institutional racism

Summary of finding	Studies contributing	Linked recommendations	Certainty of the evidence
Indigenous Peoples experiences or family experiences of racism and discrimination in the health system had generated a level of mistrust that impacted engagement with health services. For example, children being taken away from families	3 qualitative studies (5-8)	a.<br>b.	MODERATE
Cultural awareness training is inadequate and as a result the differences in management approaches with Indigenous and non-Indigenous people with CKD are not addressed	1 qualitative study (5)	a.<br>b.<br>d.	LOW
The healthcare system rigid design results in culturally inappropriate care being provided for Indigenous people. For example, management of acute care with chronic dialysis patients. Many indigenous people have experienced personally loss due to kidney failure	3 qualitative studies (5, 7, 8)	a.<br>b.	LOW

Table A2. Summary of findings – Cultural safety

Summary of finding	Studies contributing	Linked recommendations	Certainty of the evidence
Limited evaluation of cultural awareness training of healthcare staff has found improvement in knowledge and attitudes but limited evidence of changes healthcare practice	1 systematic review (9)<br>1 observational study (10)<br>1 survey evaluation (11)	a.<br>d.	LOW
Cultural awareness training was inadequate and there was a reliance on professional experience to prepare healthcare staff for providing culturally safe care	5 qualitative studies (5, 12-15)	a.<br>d.	MODERATE
Inadequate resources for healthcare staff professional development in cultural awareness/safety or its application in clinical practice	3 qualitative studies (5, 12, 15)	a.<br>d.	LOW

Table A3. Summary of findings – Person/family/community centred engagement and involvement in managing chronic kidney disease

Summary of finding	Studies contributing	Linked recommendations	Certainty of the evidence
Involving families in clinical care helped patients in decision making. Facilitating community understanding and support throughout treatment, particularly for dialysis and transplantation.	3 qualitative studies (16-18)	f.	HIGH

Table A4. Summary of findings – Transport and accommodation services for First Nations Australians

Summary of finding	Studies contributing	Linked recommendations	Certainty of the evidence
Limited access to transport and housing is a barrier during wait-listing work-up for transplantation and dialysis services	1 observational study (19) and 3 qualitative studies (7, 8, 20, 21)	g.	MODERATE
Access to housing services improved access to haemodialysis sessions in the Northern Territory	1 observational study (22)	g.<br>v.	VERY LOW
Use of community dialysis centres reduced burden on family and community and empowered people to make connection and self-manage their haemodialysis care	1 qualitative study (23)<br>1 descriptive study (24)	g.	MODERATE
Transport placed increased financial costs on families to access healthcare for children	1 systematic review (25)	g.	LOW

Table A5. Summary of findings – First Nations kidney health workforce

Summary of finding	Studies contributing	Linked recommendations	Certainty of the evidence
Limited involvement of interpreters in healthcare consultations led to lack of consideration of social determinants of health or consideration of Traditional ways to manage health of Indigenous peoples	1 qualitative study (26)	h.	MODERATE
Involvement of Aboriginal health workers improved trust and communication between patient/community and healthcare staff – Ensuring Indigenous culture was understood and considered in clinical care	4 systematic reviews (27-30)	h.	LOW
Patient navigators for Indigenous people improved health-related knowledge and adherence to screening and treatment	1 systematic review (30)	h.	LOW
Involvement of Aboriginal health workers in patient care improved knowledge, adherence to medical care, continuity of care and identification of Indigenous status	2 systematic reviews (29, 31)	h.	MODERATE

Screening and referral of chronic kidney disease

Table A6. Summary of findings - Factors associated with the incidence and progression of CKD in Indigenous peoples.

Summary of finding	Studies contributing	Linked recommendations	Certainty of the evidence
Traditional factors associated with CKD progression are evident in First Nations Australians:<br>• Diabetes<br>• High blood pressure<br>• Presence of albuminuria<br>• Higher GFR and lower BMI were found to be protective	5 observational studies (32-36)	Ungraded statement – identification of factors associated with progression of CKD	LOW
Modifiable risk factors associated with worsening kidney disease, albuminuria and lower eGFR (<60 ml/min/1.73m^2)<br>• High blood pressure<br>• Diabetes<br>• Obesity<br>• Haematuria	6 observational studies (37-42)	Ungraded statement – identification of factors associated with progression of CKD	LOW

Summary of finding	Studies contributing	Linked recommendations	Certainty of the evidence
• Older age			
Low birthweight is associated with higher albuminuria creatinine ratio and overt albuminuria (≥300 mg/g)	2 observational studies (43-45)	c.<br>Ungraded statement – identification of factors associated with progression of CKD	VERY LOW
Post-streptococcal glomerulonephritis (PSGN) has an additive impact on lower birthweight association with albuminuria	2 observational studies (44, 46)	c.<br>Ungraded statement – identification of factors associated with progression of CKD	LOW
Pre-transplant hospital admission was associated with increased graft failure among First Nation Peoples in Australia	1 Observational study (47)	c.<br>Ungraded statement – identification of factors associated with progression of CKD	VERY LOW
Higher levels of inflammation and cholesterol were associated with the presence of albuminuria	5 observational studies (32, 37-41)	Ungraded statement – identification of factors associated with progression of CKD	VERY LOW
Remoteness of Indigenous communities increased CKD prevalence and risk of CKD progression	3 observational studies (48-50)	c.<br>Ungraded statement – identification of factors associated with progression of CKD	LOW
Structural factors have been found to predict kidney function loss. However, these factors should be examined in further detail in future studies.	2 observational studies (51, 52)		VERY LOW
Genetic markers of kidney disease in Indigenous peoples have only been examined in relatively few studies and further research is required.	2 observational studies (53, 54)	c.<br>Ungraded statement – identification of factors associated with progression of CKD	VERY LOW
Racism and colonisation were identified as determinants for chronic kidney disease risk and poor outcomes in First Nations Peoples	1 systematic review (55)	c.<br>Ungraded statement – identification of factors associated with progression of CKD	LOW

Table A7. Summary of findings - Screening and early detection programmes for chronic kidney disease among First Nations Australians

Summary of finding	Studies contributing	Linked recommendations	Certainty of the evidence
Integration of screening programs into Indigenous community controlled improved uptake of screening of CKD.	4 observational studies (49, 56-58)	i.	VERY LOW
Mobile screening in remote and rural Indigenous communities improved screening for CKD and increased engagement with healthcare services	2 observational studies (59, 60)	i.<br>j.	LOW
Screening of CKD in Indigenous communities does not meet standards in guideline recommendations – frequency and coverage	1 observational study (61)	i.<br>j.	VERY LOW
One-off point of care screening was cost-effective is Indigenous communities (Screening of CKD in rural and remote Canadian Indigenous peoples with incremental cost-effectiveness ratio 23,700 CAD per QALY compared to usual care)	1 cost-effectiveness study (62)	i.<br>j.	LOW

Table A8. Summary of findings - Referral practices for First Nations Australians with chronic kidney disease

Summary of finding	Studies contributing	Linked recommendations	Certainty of the evidence
Integration of nephrology clinics within the community led by nurse-practitioners and community health workers improved identification and management of patients with CKD according to guidelines.	2 Observational studies (35, 37)	i.	VERY LOW

Public awareness, education, and self-management

Table A9. Summary of findings – Public awareness – to enable First Nations Australians to access information about kidney disease prior to screening and diagnosis

Summary of finding	Studies contributing	Linked recommendations	Certainty of the evidence
Awareness campaigns were less effective with people in lower socioeconomic quartiles	1 observational study (63)	l.	LOW
Embedding health promotion with clinical service in communities improved acceptability and trust	1 qualitative study (64)	l.<br>m.	LOW
Involvement of community members and their stories and Indigenous children improved the engagement and impact of the health promotion campaign.	1 report (65)<br>1 qualitative study (64)	l.<br>m.	VERY LOW

Table A10. Summary of findings - Education interventions that deliver knowledge to support First Nations Peoples with CKD and their community

Summary of finding	Studies contributing	Linked recommendations	Certainty of the evidence
Co-design improved education interventions utility and challenged pre-conceived ideas of CKD in community.	1 Scoping review (66)<br>1 report (67)<br>3 qualitative studies (68-70)	n.	MODERATE
Utilising preferred delivery methods of the community (Story-telling, interactive lectures) supports engagement with the community	1 Scoping review (66)<br>1 report (67)<br>3 qualitative studies (68-70)	n.<br>Ungraded statement 3 – Education	MODERATE
Lack of education about CKD, particularly during the early stages of the disease. Additionally, lack of information on the treatment options available to Indigenous people, i.e. home-based dialysis and transplantation	1 Report (71)<br>4 qualitative studies (69-72)	n.<br>Ungraded statement – identification of factors associated with progression of CKD	LOW
Education as a component of a multi-factorial intervention on managing chronic disease (diabetes, blood pressure, cholesterol) improved patient outcomes	1 observational study (73)	n.<br>Ungraded statement – Education 2	VERY LOW

Table A11. Summary of findings - Education interventions that deliver knowledge to support First Nations Peoples and their community

Summary of finding	Studies contributing	Linked recommendations	Certainty of the evidence
Co-design improved education interventions utility and challenged pre-conceived ideas of disease in the Indigenous community.	2 systematic reviews (74, 75)	n.	LOW
Practical sessions (cooking classes, self-management interventions use etc.) in group settings improved understanding of the disease and its management	1 systematic review (74)	n.<br>Ungraded statement 3 – Education	LOW

Table A12. Summary of findings table - Self-management programmes and interventions

Summary of finding	Studies contributing	Linked recommendations	Certainty of the evidence
Interventions aimed at improving self-management improved CKD related knowledge and self-management activity	5 systematic reviews (76-80)	o.<br>p.	MODERATE
Self-management and education programs aimed at self-management in dialysis patients improve interdialytic weight gain and markers of nutrition and drug adherence.	4 systematic reviews (76, 79-81)	o.<br>p.	VERY LOW
Interventions aimed at improving self-management improved some surrogate outcomes (blood pressure, HbA1c, c-reactive protein, quality of life). However, for some outcomes there were no impact (eGFR, urinary protein excretion). The long-term duration of these benefits are unclear as studies were usually of limited duration.	3 systematic review (78, 80, 82)	o.	LOW
Self-management interventions have limited evidence of improving health utilisation and hard clinical endpoints (i.e., death, kidney failure).	2 systematic reviews (78, 82)	o.	MODERATE

Models of care

Table A13. Summary of findings - Models of care – chronic kidney disease (pre-dialysis)

Summary of finding	Studies contributing	Linked recommendations	Certainty of the evidence
Health-sector led management programmes and models of care explicitly designed to manage and slow the progression of CKD, improve lives of Indigenous patients are effective at improving clinical measures	1 Systematic literature review (83)<br>1 RCT (84)<br>1 report (85)<br>1 observational study (86)	q.<br>r.	MODERATE
Programs conducted within community-controlled health services, and which utilise a multidisciplinary team approach, to implement routine CKD-specific screening measures, alongside assigning dedicated healthcare staff responsible for this screening effectively increase the identification of CKD in at-risk patients, and subsequent management	1 report (85)<br>1 observational study (86)<br>1 descriptive study (87)	q.<br>r.	LOW
Programs embedded within a community health service and involve community healthcare staff to deliver education and training to patients, effectively increase patient empowerment and quality of life, through increasing patients' knowledge and ability to self-monitor their health	1 RCT (84)	q.<br>r.	LOW
Interventions are cost-effective when utilising existing local/community resources, and when integrated within existing healthcare programs and/or initiatives	1 Systematic literature review (83)<br>1 observational study (88)<br>1 report (85)	q.<br>r.	LOW
Specialist nephrology care delivered via telehealth is a safe, acceptable and cost-effective model of care; achieving increased patient adherence to appointments, retention, as well as provides numerous additional benefits to patients (reduced travel distance, greater independence, improved satisfaction)	1 observational study (88)	s.<br>t.	LOW
Co-design and involvement of Indigenous people, healthcare workers, and community services, alongside inter- and multi-disciplinary integration facilitates implementation	1 systematic literature review (83)<br>1 RCT (84)<br>2 observational studies (86, 88)<br>1 report (85)<br>1 descriptive study (87)	q.<br>r.	MODERATE

Summary of finding	Studies contributing	Linked recommendations	Certainty of the evidence
Establishing governance and service structures, including utilisation of, and collaboration within existing care delivery systems facilitates implementation	1 systematic literature review (83)<br>1 descriptive study (87)<br>1 report (85)	s.	MODERATE
Clinical care and education programs that are culturally-appropriate, tailored and flexible facilitate implementation	1 systematic literature review (83)<br>1 RCT (84)<br>1 observational study (88)<br>1 descriptive study (87)<br>1 report (85)	r.<br>t.	MODERATE
Clinical and medical information systems (technology, programming, documentation) that are able to support program delivery, alongside quality improvement tools are integral to facilitating effective program implementation	1 systematic literature review (83)<br>1 descriptive study (87)<br>1 report (85)	s.	MODERATE
Promotion of a health service's mission and workplace culture, and support of the multidisciplinary healthcare staff (provision of continuing education programs, case management workshops, clinic guidelines/protocols, online education materials) to deliver the intervention program facilitated program efficacy and success	2 reports (85, 89)	r.<br>t.	LOW
Barriers to program implementation include not addressing (cost, lack of transport), exclusion of Indigenous peoples and perspectives from decision-making, poorly performing electronic support systems	1 systematic literature review (83)<br>1 observational study (88)	r.<br>s.<br>t.	MODERATE

Table A14. Summary of findings - Models of care (Kidney failure)

Summary of findings	Studies contributing	Linked recommendations	Certainty of evidence
Community controlled, nurse supported, remote area dialysis models are clinically safe, with no mortality disadvantage, and allow people to have dialysis on Country.	Clinical effectiveness	u.<br>w.	VERY LOW
	Marley 2010 (90)		
Community controlled, nurse supported, remote area dialysis models are cost effective when total health expenditure is considered.	Cost effectiveness	u.<br>w.	LOW
	Gorham 2021 (91)		
	Gorham 2019 (92)		
Urban community-based home haemodialysis is a safe model, which does not increase mortality when compared to conventional home haemodialysis.	Clinical effectiveness	u.<br>y.	VERY LOW
	Marshall 2013 (93)		
	Brown 2010 (94)		
Urban community-based home haemodialysis is acceptable to First Nations communities and may reduce the treatment burden of in-centre dialysis.	Patient/staff acceptability	u.<br>y.	VERY LOW
	Walker 2019 (23)		
Adopting a primary care supported, telehealth model of dialysis care does not lead to health disadvantage in term of clinical outcomes or mortality. Telehealth should be considered as an option to augment traditional face-to-face care.	Clinical effectiveness	z.	VERY LOW
	Sicotte 2011 (95)		
Mobile dialysis units may facilitate return to Country and have important psychosocial benefits for patients and community. However, successful uptake of mobile dialysis requires local accommodation, transport, and sufficient workforce support.	Patient/staff acceptability	v.	VERY LOW
	Conway 2018 (96)		
Ensuring stable, long term accommodation options can increase engagement with treatment and may increase dialysis attendance	Clinical effectiveness	g.<br>v.	VERY LOW
	Ahmed 2018 (22)		

References

1. Rix EF, Barclay L, Wilson S, Stirling J, Tong A. Service providers' perspectives, attitudes and beliefs on health services delivery for Aboriginal people receiving haemodialysis in rural Australia: A qualitative study. BMJ Open. 2013;3(10).
2. Rix EF, Barclay L, Stirling J, Tong A, Wilson S. The perspectives of Aboriginal patients and their health care providers on improving the quality of hemodialysis services: A qualitative study. Hemodial Int. 2015;19(1):80-9.
3. Walker RC, Abel S, Palmer SC, Walker C, Heays N, Tipene-Leach D. Values, Perspectives, and Experiences of Indigenous Māori Regarding Kidney Transplantation: A Qualitative Interview Study in Aotearoa/New Zealand. Am J Kidney Dis. 2022.
4. Walker RC, Abel S, Palmer SC, Walker C, Heays N, Tipene-Leach D. "We Need a System that's Not Designed to Fail Māori": Experiences of Racism Related to Kidney Transplantation in Aotearoa New Zealand. J Racial Ethn Health Disparities. 2022:1-9.
5. Clifford A, McCalman J, Bainbridge R, Tsey K. Interventions to improve cultural competency in health care for Indigenous peoples of Australia, New Zealand, Canada and the USA: A systematic review. International Journal for Quality in Health Care. 2015;27(2):89-98.
6. Barnabe C, Kherani RB, Appleton T, Umaefulam V, Henderson R, Crowshoe L. Participant-reported effect of an Indigenous health continuing professional development initiative for specialists. BMC Medical Education. 2021;21(1):116.
7. Abbott P, Reath J, Gordon E, Dave D, Harnden C, Hu W, et al. General Practitioner Supervisor assessment and teaching of Registrars consulting with Aboriginal patients - is cultural competence adequately considered? BMC Med Educ. 2014;14:167.
8. Downing R, Kowal E. Putting Indigenous cultural training into nursing practice. Contemporary nurse : a journal for the Australian nursing profession. 2011;37(1):10-20.
9. Kerrigan V, Lewis N, Cass A, Hefler M, Ralph AP. "How can I do more?" Cultural awareness training for hospital-based healthcare providers working with high Aboriginal caseload. BMC Medical Education. 2020;20(1):173.
10. Watt K, Abbott P, Reath J. Cultural competency training of GP Registrars-exploring the views of GP Supervisors. Intern. 2015;14:89.
11. Withall L, Ryder C, Mackean T, Edmondson W, Sjoberg D, McDermott D, et al. Assessing cultural safety in Aboriginal and Torres Strait Islander Health. Aust J Rural Health. 2021;29(2):201-10.
12. Devitt J, Anderson K, Cunningham J, Preece C, Snelling P, Cass A. Difficult conversations: Australian Indigenous patients' views on kidney transplantation. BMC Nephrology. 2017;18(1):310.
13. Rix EF, Barclay L, Stirling J, Tong A, Wilson S. The perspectives of Aboriginal patients and their health care providers on improving the quality of hemodialysis services: a qualitative study. Hemodial. 2015;19(1):80-9.
14. Walker RC, Walker S, Morton RL, Tong A, Howard K, Palmer SC. Maori patients' experiences and perspectives of chronic kidney disease: a New Zealand qualitative interview study. BMJ Open. 2017;7(1):e013829.
15. Majoni SW, Dole K, Hughes JT, Pain C. Review of current pathways to wait-listing for kidney transplantation for Aboriginal and Torres Strait Islander peoples with end-stage kidney disease in the Top End of Northern Australia. Australian health review : a publication of the Australian Hospital Association. 2021;45(2):185-93.
16. Scholes-Robertson N, Blazek K, Tong A, Craig J, Essue B, Howard K, et al. The Financial burden of chronic kidney disease for rural Australian families [Abstract]. Nephrology. 2021;26:36-.
17. Walker RC, Hay S, Walker C, Tipene-Leach D, Palmer SC. Exploring rural and remote patients' experiences of health services for kidney disease in Aotearoa New Zealand: An in-depth interview study. Nephrology. 2022;27(5):421-9.

18. Ahmed S, Lawton P, Gorham G, Hughes J, O'Hehir C, Francis C, et al. Housing provision and health outcomes among northern territory haemodialysis patients: The dialysis models of care partnership. Nephrology. 2018;23(Supplement 3):26.
19. Walker RC, Tipene-Leach D, Graham A, Palmer SC. Patients' Experiences of Community House Hemodialysis: A Qualitative Study. Kidney Med. 2019;1(6):338-46.
20. Shashi bhaskara S, Cherian S, Pawar B, Fernandes D, Sureshkumar R, Gosling C. Respite hemodialysis to remote indigenous central australian population and mobile dialysis unit. Nephrology. 2012;2):47.
21. Coombes J, Hunter K, Mackean T, Holland AJA, Sullivan E, Ivers R. Factors that impact access to ongoing health care for First Nation children with a chronic condition. BMC Health Services Research. 2018;18(1):448.
22. Cass A, Lowell A, Christie M, Snelling PL, Flack M, Marrnganyin B, et al. Sharing the true stories: improving communication between Aboriginal patients and healthcare workers. Medical Journal of Australia. 2002;176(10):466-70.
23. Ehrlich C, Kendall E, Parekh S, Walters C. The impact of culturally responsive self-management interventions on health outcomes for minority populations: A systematic review. Chronic Illn. 2016;12(1):41-57.
24. Gomersall JS, Gibson O, Dwyer J, O'Donnell K, Stephenson M, Carter D, et al. What Indigenous Australian clients value about primary health care: a systematic review of qualitative evidence. Aust N Z J Public Health. 2017;41(4):417-23.
25. Mackean T, Withall E, Dwyer J, Wilson A. Role of Aboriginal Health Workers and Liaison Officers in quality care in the Australian acute care setting: a systematic review. Australian health review : a publication of the Australian Hospital Association. 2020;44(3):427-33.
26. Whop LJ, Valery PC, Beesley VL, Moore SP, Lokuge K, Jacka C, et al. Navigating the cancer journey: a review of patient navigator programs for Indigenous cancer patients. Asia Pac J Clin Oncol. 2012;8(4):e89-96.
27. Chang AB, Taylor B, Masters IB, Laifoo Y, Brown AD. Indigenous healthcare worker involvement for Indigenous adults and children with asthma. Cochrane Database of Systematic Reviews. 2010(5):CD006344.
28. Afshinnia F, Nair V, Lin J, Rajendiran TM, Soni T, Byun J, et al. Increased lipogenesis and impaired B-oxidation predict type 2 diabetic kidney disease progression in American Indians. JCI Insight. 2019;4(21).
29. Hoy WE, Wang Z, VanBuynder P, Baker PR, McDonald SM, Mathews JD. The natural history of renal disease in Australian Aborigines. Part 2. Albuminuria predicts natural death and renal failure. Kidney Int. 2001;60(1):249-56.
30. Maple-Brown LJ, Hughes JT, Ritte R, Barzi F, Hoy WE, Lawton PD, et al. Progression of kidney disease in indigenous Australians: The eGFR follow-up study. Clinical Journal of the American Society of Nephrology. 2016;11(6):993-1004.
31. Ritte R, Luke J, Nelson C, Brown A, O'Dea K, Jenkins A, et al. Clinical outcomes associated with albuminuria in central Australia: a cohort study. BMC Nephrology. 2016;17(1):113.
32. Chilumkurti V, Zhang J, Wang Z, Diwan V, Hoy W. Chronic kidney disease and comorbidity in Indigenous Communities in Australia. Nephrology. 2021;26:19-.
33. Dart AB, Wicklow B, Blydt-Hansen TD, Sellers EAC, Malik S, Chateau D, et al. A Holistic Approach to Risk for Early Kidney Injury in Indigenous Youth With Type 2 Diabetes: A Proof of Concept Paper From the iCARE Cohort. Can J Kidney Health Dis. 2019;6(no pagination).
34. Dyck RF, Hayward MN, Harris SB, Group CS. Prevalence, determinants and co-morbidities of chronic kidney disease among First Nations adults with diabetes: results from the CIRCLE study. BMC Nephrology. 2012;13:57.
35. Hughes JT, Maple-Brown LJ, Thomas M, Lawton PD, Sinha A, Cass A, et al. Cross-sectional associations of albuminuria among Aboriginal and Torres Strait Islander adults: the eGFR Study. Nephrology. 2018;23(1):37-45.

36. Jolly SE, Li S, Chen SC, Narva AS, Jurkovitz CT, Norris KC, et al. Risk factors for chronic kidney disease among American Indians and Alaska Natives - Findings from the kidney early evaluation program. Am J Nephrol. 2009;29(5):440-6.
37. Maple-Brown LJ, Cunningham J, Hodge AM, Weeramanthri T, Dunbar T, Lawton PD, et al. High rates of albuminuria but not of low eGFR in urban indigenous Australians: the DRUID study. BMC Public Health. 2011;11:346.
38. Shephard MD, Allen GG, Barratt LJ, Barbara JA, Paizis K, McLeod G, et al. Albuminuria in a remote South Australian Aboriginal community: results of a community-based screening program for renal disease. Rural Remote Health. 2003;3(1):156.
39. Hoy WE, Rees M, Kile E, Mathews JD, Wang Z. A new dimension to the Barker hypothesis: low birthweight and susceptibility to renal disease. Kidney Int. 1999;56(3):1072-7.
40. Hoy WE, White AV, Tipiloura B, Singh GR, Sharma S, Bloomfield H, et al. The influence of birthweight, past poststreptococcal glomerulonephritis and current body mass index on levels of albuminuria in young adults: The multideterminant model of renal disease in a remote Australian Aboriginal population with high rates of renal disease and renal failure. Nephrol Dial Transplant. 2016;31(6):971-7.
41. Hoy WE, Swanson CE, Mott SA. Birthweight and the Prevalence, Progression, and Incidence of CKD in a Multideterminant Model in a High-Risk Australian Aboriginal Community. Kidney Int Rep. 2021;6(11):2782-93.
42. White AV, Hoy WE, McCredie DA. Childhood post-streptococcal glomerulonephritis as a risk factor for chronic renal disease in later life. Medical Journal of Australia. 2001;174(10):492-6.
43. Khanal N, Lawton PD, Cass A, McDonald SP. A retrospective case-control study exploring pre-transplant predictors for loss of kidney transplant function or death among Indigenous kidney transplant recipients. Intern Med J. 2021.
44. Harasemiw O, Milks S, Oakley L, Lavallee B, Chartrand C, McLeod L, et al. Remote Dwelling Location Is a Risk Factor for CKD Among Indigenous Canadians. Kidney Int Rep. 2018;3(4):825-32.
45. Komenda P, Lavallee B, Ferguson TW, Tangri N, Chartrand C, McLeod L, et al. The Prevalence of CKD in Rural Canadian Indigenous Peoples: Results From the First Nations Community Based Screening to Improve Kidney Health and Prevent Dialysis (FINISHED) Screen, Triage, and Treat Program. American Journal of Kidney Diseases. 2016;68(4):582-90.
46. Ritte RE, Lawton P, Hughes JT, Barzi F, Brown A, Mills P, et al. Chronic kidney disease and socio-economic status: a cross sectional study. Ethn Health. 2020;25(1):93-109.
47. Fufaa GD, Weil EJ, Lemley KV, Knowler WC, Brosius FC, 3rd, Yee B, et al. Structural Predictors of Loss of Renal Function in American Indians with Type 2 Diabetes. Clin J Am Soc Nephrol. 2016;11(2):254-61.
48. Barr E, Hughes JT, Barzi F, Obeyesekere V, Hoy W, O'Dea K, et al. Novel markers and prediction of kidney disease progression in Aboriginal and Torres Strait Isladner People with and without diabetes: the eGFR study. Nephrology (Carlton). 2021;26:33-.
49. Hoy W, Jadhao S, Thomson R, Mathews J, Patel C, Andrew M, et al. Sat-191 Whole Genome Analysis of Aboriginal Australians Reveals Variants Associated with Kidney Disease. Kidney International Reports. 2019;4 (7 Supplement):S87-S8.
50. McDonald SP, Hoy WE, Maguire GP, Duarte NL, Wilcken DE, Wang XL. The p53Pro72Arg polymorphism is associated with albuminuria among aboriginal Australians. J Am Soc Nephrol. 2002;13(3):677-83.
51. Huria T, Pitama SG, Beckert L, Hughes J, Monk N, Lacey C, et al. Reported sources of health inequities in Indigenous Peoples with chronic kidney disease: a systematic review of quantitative studies. BMC Public Health. 2021;21(1):1447.
52. Ashton CW, Duffie D. Chronic kidney disease in Canada's First Nations: results of an effective cross-cultural collaboration. Healthc Q. 2011;14(3):42-7.

53. Barrett E, Salem L, Wilson S, O'Neill C, Davis K, Bagnulo S. Chronic kidney disease in an Aboriginal population: A nurse practitioner-led approach to management. Aust J Rural Health. 2015;23(6):318-21.
54. Gorham G, Chatfield M, Lawton P, Cass A. Analysis of longitudinal clinical data to evaluate changes in the screening for and management of CKD in select NT primary health services. Nephrology. 2017;22 (Supplement 3):65.
55. Frejuk KL, Harasemiw O, Komenda P, Lavallee B, McLeod L, Chartrand C, et al. Impact of a screen, triage and treat program for identifying chronic disease risk in Indigenous children. Can Med Assoc J. 2021;193(36):E1415-e22.
56. Harasemiw O, Ferguson T, Lavallee B, McLeod L, Chartrand C, Rigatto C, et al. Impact of point-of-care screening for hypertension, diabetes and progression of chronic kidney disease in rural Manitoba Indigenous communities. Can Med Assoc J. 2021;193(28):E1076-e84.
57. Berghout M, Page W. Screening and staging of chronic kidney disease: An audit in aboriginal people of the gove region in 2011-2. Nephrology. 2013;18:46.
58. Ferguson TW, Tangri N, Tan Z, James MT, Lavallee BDA, Chartrand CD, et al. Screening for chronic kidney disease in Canadian indigenous peoples is cost-effective. Kidney Int. 2017;92(1):192-200.
59. Ryz K, Tangri N, Verrelli M, Schneider J, Lesyk A, Eng A, et al. A before and after cross-sectional analysis of a public health campaign to increase kidney health awareness in a Canadian province. BMC Research Notes. 2015;8:695.
60. Sinclair C, Stokes A, Jeffries-Stokes C, Daly J. Positive community responses to an arts-health program designed to tackle diabetes and kidney disease in remote Aboriginal communities in Australia: a qualitative study. Aust N Z J Public Health. 2016;40(4):307-12.
61. Britt RK, Britt BC, Anderson J, Fahrenwald N, Harming S. "Sharing Hope and Healing": A Culturally Tailored Social Media Campaign to Promote Living Kidney Donation and Transplantation Among Native Americans. Health Promot Pract. 2020:1524839920974580.
62. Jansen L, Maina G, Horsburgh B, Kumaran M, McHaro K, Laliberte G, et al. Co-Developed Indigenous Educational Materials for Chronic Kidney Disease: A Scoping Review. Canadian Journal of Kidney Health and Disease. 2020;7(no pagination).
63. Thomas C. Development of a culturally sensitive, locality-based program to increase kidney donation. Advances in Renal Replacement Therapy. 2002;9(1):54-6.
64. Cotsianis L, Lindsay A, Plamondon J. Renal health outreach (RHO) in Manitoba: Opportunities for improvement. American Journal of Kidney Diseases. 2011;57 (4):A34.
65. Richels L, Sutherland W, Prettyshield K, Kappel J, McCarron M, Richardson B. Community-Based Dialysis in Saskatchewan First Nations: A Grassroots Approach to Gaining Insight and Perspective From First Nations Patients With Chronic Kidney Disease. Can J Kidney Health Dis. 2020;7:2054358120914689.
66. Rix EF, Barclay L, Stirling J, Tong A, Wilson S. 'Beats the alternative but it messes up your life': aboriginal people's experience of haemodialysis in rural Australia. BMJ Open. 2014;4(9):e005945.
67. Keddis M, Finnie D, Kim WS. Native American patients' perception and attitude about kidney transplant: a qualitative assessment of patients presenting for kidney transplant evaluation. BMJ Open. 2019;9(1):e024671.
68. Jolly SE, Haring RC, O'Leary M, Sehgal A, Manson S, Killion C. Understanding kidney health and chronic kidney disease (CKD) knowledge among American Indians: A focus group study. J Gen Intern Med. 2016;31(2):S445-S6.
69. Hoy WE, Baker PR, Kelly AM, Wang Z. Reducing premature death and renal failure in Australian aboriginals. A community-based cardiovascular and renal protective program. Medical Journal of Australia. 2000;172(10):473-8.

70. McCalman J, Tsey K, Wenitong M, Wilson A, McEwan A, James YC, et al. Indigenous men's support groups and social and emotional wellbeing: a meta-synthesis of the evidence. Aust J Prim Health. 2010;16(2):159-66.
71. Schembri L, Curran J, Collins L, Pelinovskaia M, Bell H, Richardson C, et al. The effect of nutrition education on nutrition-related health outcomes of Aboriginal and Torres Strait Islander people: a systematic review. Aust N Z J Public Health. 2016;40 Suppl 1:S42-7.
72. Cho MK, Kang Y. Effect of self-care intervention for controlling interdialytic weight gain among patients on haemodialysis: A systematic review and meta-analysis. Journal of Clinical Nursing. 2021;30(15-16):2348-65.
73. Donald M, Kahlon BK, Beanlands H, Straus S, Ronksley P, Herrington G, et al. Self-management interventions for adults with chronic kidney disease: a scoping review. BMJ Open. 2018;8(3):e019814.
74. Zimbudzi E, Lo C, Misso ML, Ranasinha S, Kerr PG, Teede HJ, et al. Effectiveness of self-management support interventions for people with comorbid diabetes and chronic kidney disease: a systematic review and meta-analysis. Syst Rev. 2018;7(1):84.
75. Reid C, Hall J, Boys J, Lewis S, Chang A. Self management of haemodialysis for End Stage Renal Disease: a systematic review. JBI Evidence Synthesis. 2011;9(3).
76. Lin MY, Liu MF, Hsu LF, Tsai PS. Effects of self-management on chronic kidney disease: A meta-analysis. International Journal of Nursing Studies. 2017;74:128-37.
77. Yangoz ST, Ozer Z, Boz I. Comparison of the effect of educational and self-management interventions on adherence to treatment in hemodialysis patients: A systematic review and meta-analysis of randomized controlled trials. International Journal of Clinical Practice. 2021;75(5):e13842.
78. Peng S, He J, Huang J, Lun L, Zeng J, Zeng S, et al. Self-management interventions for chronic kidney disease: a systematic review and meta-analysis. BMC Nephrology. 2019;20(1):142.
79. Reilly R, Evans K, Gomersall J, Gorham G, Peters MD, Warren S, et al. Effectiveness, cost effectiveness, acceptability and implementation barriers/enablers of chronic kidney disease management programs for Indigenous people in Australia, New Zealand and Canada: a systematic review of mixed evidence. BMC Health Services Research. 2016;16(1):1-15.
80. Nelson RG, Pankratz VS, Ghahate DM, Bobelu J, Faber T, Shah VO. Home-based kidney care, patient activation, and risk factors for CKD progression in Zuni Indians: a randomized, controlled clinical trial. Clinical Journal of the American Society of Nephrology. 2018;13(12):1801-9.
81. Narva AS. Reducing the burden of chronic kidney disease among American Indians. Advances in chronic kidney disease. 2008;15(2):168-73.
82. Gorham G, Chatfield M, Lawton P, Cass A. Analysis of longitudinal clinical data to evaluate changes in the screening for and management of CKD in select NT primary health services. Nephrology. 2017;22(Supplement 3):65.
83. Barrett E, Salem L, Wilson S, O'Neill C, Davis K, Bagnulo S. Chronic kidney disease in an Aboriginal population: A nurse practitioner-led approach to management. Aust J Rural Health. 2015;23(6):318-21.
84. Venuthurupalli SK, Rolfe A, Fanning J, Cameron A, Hoy WE. Chronic Kidney Disease, Queensland (CKD. QLD) registry: management of CKD with telenephrology. Kidney Int Rep. 2018;3(6):1336-43.
85. Narva A. Population health for CKD and diabetes: lessons from the Indian Health Service. American Journal of Kidney Diseases. 2018;71(3):407-11.
86. Marley JV, Dent HK, Wearne M, Fitzclarence C, Nelson C, Siu K, et al. Haemodialysis outcomes of Aboriginal and Torres Strait Islander patients of remote Kimberley region origin. Med J Aust. 2010;193(9):516-20.
87. Gorham G, Howard K, Cunningham J, Barzi F, Lawton P, Cass A. Do remote dialysis services really cost more? An economic analysis of hospital and dialysis modality costs associated

with dialysis services in urban, rural and remote settings. BMC Health Services Research. 2021;21(1):582.
88. Gorham G, Howard K, Zhao Y, Ahmed AMS, Lawton PD, Sajiv C, et al. Cost of dialysis therapies in rural and remote Australia - a micro-costing analysis. BMC Nephrol. 2019;20(1):231.
89. Marshall MR, van der Schrieck N, Lilley D, Supershad SK, Ng A, Walker RC, et al. Independent community house hemodialysis as a novel dialysis setting: an observational cohort study. American Journal of Kidney Diseases. 2013;61(4):598-607.
90. Brown D, Gorham G, Barnes J. Setting up home hemodialysis in remote communities. Clinical Nephrology. 2010;74:S162-S3.
91. Sicotte C, Moqadem K, Vasilevsky M, Desrochers J, St-Gelais M. Use of telemedicine for haemodialysis in very remote areas: The Canadian first nations. Journal of Telemedicine and Telecare. 2011;17(3):146-9.
92. Conway J, Lawn S, Crail S, McDonald S. Indigenous patient experiences of returning to country: a qualitative evaluation on the Country Health SA Dialysis bus. BMC Health Services Research. 2018;18(1):N.PAG-N.PAG.
